# Hydraulic conductivity and contribution of aquaporins to water uptake in roots of four sunflower genotypes

**DOI:** 10.1186/s40529-014-0075-1

**Published:** 2014-10-30

**Authors:** Afifuddin Latif Adiredjo, Olivier Navaud, Philippe Grieu, Thierry Lamaze

**Affiliations:** 1 Université de Toulouse, INP - ENSAT, UMR 1248 AGIR (INPT-INRA), Castanet-Tolosan, 31326 France; 2 Faculty of Agriculture, Department of Agronomy, Plant Breeding Laboratory, Brawijaya University, Veteran street, Malang, 65145 Indonesia; 3 Université de Toulouse, UPS - Toulouse III, UMR 5126 CESBIO, 18 avenue Edouard Belin, Toulouse, 31401 Cedex 9 France

**Keywords:** Sunflower, Aquaporins, Root, Hydraulic conductivity

## Abstract

**Background:**

This article evaluates the potential of intraspecific variation for whole-root hydraulic properties in sunflower. We investigated genotypic differences related to root water transport in four genotypes selected because of their differing water use efficiency (JAC doi: 10.1111/jac.12079. 2014). We used a pressure-flux approach to characterize hydraulic conductance (*L*_*0*_) which reflects the overall water uptake capacity of the roots and hydraulic conductivity (*Lp*_*r*_) which represents the root intrinsic water permeability on an area basis. The contribution of aquaporins (AQPs) to water uptake was explored using mercuric chloride (HgCl_2_), a general AQP blocker.

**Results:**

There were considerable variations in root morphology between genotypes. Mean values of *L*_*0*_ and *Lp*_*r*_ showed significant variation (above 60% in both cases) between recombinant inbred lines in control plants. Pressure-induced sap flow was strongly inhibited by HgCl_2_ treatment in all genotypes (more than 50%) and contribution of AQPs to hydraulic conductivity varied between genotypes. Treated root systems displayed markedly different *L*_*0*_ values between genotypes whereas *Lp*_*r*_ values were similar.

**Conclusions:**

Our analysis points to marked differences between genotypes in the intrinsic aquaporin-dependent path (*Lp*_*r*_ in control plants) but not in the intrinsic AQP-independent paths (*Lp*_*r*_ in HgCl_2_ treated plants). Overall, root anatomy was a major determinant of water transport properties of the whole organ and can compensate for a low AQP contribution. Hydraulic properties of root tissues and organs might have to be taken into account for plant breeding since they appear to play a key role in sunflower water balance and water use efficiency.

**Electronic supplementary material:**

The online version of this article (doi:10.1186/s40529-014-0075-1) contains supplementary material, which is available to authorized users.

## Background

Terrestrial plants are dependent on essential leaf physiological processes such as a continuous supply with water since photosynthesis cannot be dissociated from transpiration. Water balance at the whole plant level should be regulated by coupled responses between the above-ground and below-ground parts (Shimizu et al. [2005]). In the soil-plant-atmosphere continuum, the root offers the second largest resistance to water transport after the stomata (Steudle et al. [1987]). Thus, gaining information on the hydraulic properties of roots might be a key step for understanding whole-plant water relations and selection of water stress-resistant species or genotypes and therefore plant breeding (Sutka et al. [2011]).

In roots in which the xylem vessels are fully developed, the resistance to water transport occurs radially (Steudle and Peterson [1998]; Ruggiero et al. [2003]). Radial transport in roots crosses both the cell-to-cell pathway and the apoplastic pathway. The cell-to-cell route is composed of the symplastic (through the plasmodesmata) and the transcellular (involving crossing of membranes) paths. The apoplastic pathway is usually considered to have the least hydraulic resistance and is often considered to be the main route (Heinen et al. [2009]). However, the presence of lignified or suberized cell walls (casparian strips in root endodermis) which constitute apoplastic barriers forces water to cross cell membranes (Shimizu et al. [2005]). Several studies have attributed an important role to the cell-to-cell path. Water movement through cell membranes is facilitated by water channels, called “aquaporins” (AQPs) (Maurel [2007]). AQPs are integral membrane proteins that increase the permeability of membranes to water as well as other small molecules such as CO_2_, glycerol and boron (Shimizu et al. [2005]; Chaumont et al. [2005]). AQP proteins contain thiol groups that are sensitive to HgCl_2_ (Savage and Stroud [2007]). Assuming that mercurial inhibition of water transport occurs via the inhibition of AQPs, the strength of inhibition may indicate the extent to which the cell-to-cell (transcellular) water movement (involving water passing through membranes) is involved in the radial transport of water across the root. Therefore, to divide radial water transport in roots into cell-to-cell and apoplastic pathways, HgCl_2_ has often been used as a specific AQP inhibitor in crops, herbs and trees (Maggio and Joly [1995]; Carvajal et al. [1996]; Tazawa et al. [1997]; Zhang and Tyerman [1999]; Wan and Zwiazek [1999]; North et al. [2004]; Kamaluddin and Zwiazek [2001]; Shimizu et al. [2005]; Sutka et al. [2011]).

Intraspecific root water transport has so far been compared in only a small number of species: rice, maize, grapevine and *Arabidopsis* (Sutka et al. [2011]). Sunflower is an economically important crop consumed worldwide. Although it is considered to be relatively tolerant to water stress, sunflower production can be greatly affected by drought (Pasda and Diepenbrock [1990]; Grieu et al. [2008]). Indeed, although sunflower extracts water efficiently and then conducts it within the plant, rates of leaf transpiration can reach very high values: up to 22 mmol H_2_O m^−2^s^−1^ (Rawson et al. [1980]). It has been shown that AQPs play a role in the sunflower response to drought in both the leaf and the root (Ouvrard et al. [1996]; Sarda et al. [1997], [1999]). Information on the hydraulic properties of roots might be important for plant breeding. Thus, the aim of the present work was to evaluate the variations in the root hydraulic properties of four sunflower genotypes selected because of their differing whole-plant water relations under well-watered conditions (Adiredjo et al. [2014]). We used pressure-induced flow through root systems since the method has been widely employed to measure the hydraulic properties of roots in various plant species. The contribution of the AQP-dependent pathway (cell-to-cell route) to water transport was characterized using mercuric chloride (mercury) as an inhibitor.

## Methods

### Plant source

Four recombinant inbred lines (RILs) of sunflower (*Helianthus annuus* L.) from the collection of the Laboratory of Plant-Microbe Interactions (LIPM), INRA of Toulouse, France, were used in the experiments, namely: RIL 043, RIL 127, RIL 149 and RIL 200. The four RILs are lines from the INEDI RIL population. This population was obtained by self pollination to at least F8 from a cross between XRQ and PSC8 (Vincourt et al. [2012]). These parental lines have different drought tolerance behavior (Rengel et al. [2012]). The four RILs were chosen on the basis of their differing water use efficiency (WUE) response under well-watered conditions (Adiredjo et al. [2014]).

### Plant culture, experimental design and root analysis

The plants were grown in a growth chamber (25°C/20°C in day/night) under 14 h of light (200 μmol m^−2^ s^−1^ photosynthetically active radiation at leaf level, Fluora, L 58 W/77, Germany) and 50 ± 5% RH in 250 mL pots. They were arranged in a randomized complete design with four RILs. To minimize the effects of heterogeneity within the growth chamber, the pots were rotated every week. Root hydraulics parameters (conductance, conductivity and contribution of AQPs) were measured by determining pressure-induced sap rates in six-week-old sunflower seedlings, when above-ground parts were 15–20 cm high. The experiment was repeated three times.

Seedlings were grown in 250 mL glass pots filled with sand which could be easily washed and saturated with solution and then introduced into the pressure chamber. Thus the root system did not have to be excavated before the pressure-induced flow experiment and thereafter, excavation of the roots from the cultivated sand substrate could be gently achieved under water for determination of root characteristics. Upon completion of the exudation experiments, root fresh weight was determined. Then, the properties i.e. root length, root surface area and volume of fine roots of each root system were determined with an image analyzer WinRHIZO 2007d (Regent Instruments, Quebec, Canada). Fine roots are the smallest diameter class (0 – 0.5 mm). Finally, root dry weight was measured.

### Measurement of root sap flow and HgCltreatment

For pressure-flow experiments, upon harvest, pots were washed three times (3 ± 50 mL) to saturation with water for the control treatment and HgCl_2_ solution (500 μM) for the inhibited treatment. Saturation of the pots allowed us to determine root conductivity since under non-limiting soil moisture, plant resistance exceeds soil resistance (in wet soil, the bulk soil potential is close to 0 MPa, Ruggiero et al. [1999]; [2003]). Following this washing, the above-ground part was cut off with a razor blade just below the cotyledonary leaves (40–50 mm from the base). Pots with whole root systems were placed in a stainless steel pressure chamber (Soil Moisture Equipment Corp., Santa Barbara, CA, USA, Maggio and Joly [1995]). Excised stems were sealed into the lid of the chamber through a silicone gasket so that part of the stem protruded and chamber pressure was gradually increased. Water expressed from each cut stem was collected using an Eppendorf tube containing dry cotton wool. The amount of sap was determined by weighing the tube before and after collection. The sap flow (Jv), expressed as the quantity of water exuded from the cut stems, was monitored every 5 min for at least 45 min after it had reached a constant rate (reached in less than 25 min). Aliquots of expressed sap were collected from each root system for later analysis of K^+^ content.

In preliminary experiments (pressure-flux curves), five pressures were applied in sequentially increasing order (0.1, 0.2, 0.3, 0.4 and 0.5 MPa) to whole root systems. Flow values were logged for 25 min at each pressure, allowing a 5-min equilibration period between pressures. Because the regression of sap flow on applied pressure was linear for all genotypes (data not shown), sap flows in both control and HgCl_2−_treated plants were determined at a constant 0.3 MPa pressure (Shimizu et al. [2005]). Pressure was gradually increased up to 0.3 MPa in the chamber, and was then held constant during the measurements (flow reached a steady-state value in about 20 min). The sap flow (Jv) was then used to define (i) the whole root hydraulic conductance (*L*_*0*_) calculated as the sap flow rate per unit pressure (μL s^−1^ MPa^−1^) and (ii) the root hydraulic conductivity (*Lp*_*r*_) calculated as the sap flow rate per unit root surface area and per unit pressure (m s^−1^ MPa^−1^).

We performed preliminary experiments to determine the more suitable of two methods to treat the plants with the HgCl_2_ inhibitor. First, after measurement of the pressure that induced sap flow in untreated roots, the pressure was released slowly before opening the chamber, the cut stem was removed from the gasket and the pot was flushed with HgCl_2_ solution. The stem was once more sealed in the gasket secured to the lid and the pressure in the chamber again set to 0.3 MPa. However, this was a delicate procedure that often caused damage to the stem. Second, measurement of the pressure that induced sap flow was done on distinct root systems, untreated and treated (control and HgCl_2_). Therefore, calculation of the depressive effect of HgCl_2_ on Jv was finally achieved by considering the second method rather than the first since both gave similar results. Experiments were performed three times using three plants per RIL each time.

After flushing the pot with HgCl_2_, maximal inhibition was achieved in less than 40 min. The reversibility of inhibition by HgCl_2_ of pressure-induced sap flow was evaluated by flushing of the pot with 3×50 mL 10 mM mercaptoethanol (ME) . The decrease brought about by HgCl_2_ was reversed by *ca* 90% on subsequent treatment for 30 min with ME. Some sap samples collected from control or HgCl_2_-treated de-topped plants were diluted (*ca* 40 μL of sap +1 mL H_2_O) and injected into a Dionex-D-100 ion chromatograph (USA). K^+^ flux through the xylem was calculated as the product of the sap flux and concentration of K^+^ in the sap.

### Statistics

Data were analyzed with the PASW statistics 18 (IBM, New York, USA) package. We used the Least Significant Difference (LSD) test (*P* <0.05) to make post-hoc comparisons between all means. Percentages of inhibition by HgCl_2_ were calculated for each individual root system, and mean values and standard deviations were calculated for the three experiments. In total, the response of nine HgCl_2_-treated plants was compared with those of untreated plants for each genotype.

## Results

### Size of root systems

Morphological parameters of sunflower root systems are presented in Table 1. RIL 043 and RIL 127 had the largest and the smallest root systems for every parameter (fresh or dry mass, surface, length and fine root volume), respectively. Differences reached up to 100% of the values, demonstrating considerable variation in root morphology between genotypes. RIL 149 and RIL 200 showed intermediate root characteristics with the ranking: RIL149 > RIL200.

**Table 1:** **Root morphological parameters and K** ^**+**^ **flux into the xylem of four sunflower genotypes grown in a growth chamber under controlled and well-watered conditions**

Genotype	***N***	RFW (g)	RDW (g)	RL (cm)	RS (cm^2^)	RV (cm^3^)^a^	K^+^flux (μmol h^−1^g^−1^)
RIL043	23	7.92 ±2.08	0.29 ± 0.06	1669 ± 331	210 ± 35	2.39 ± 0.74	5.08 ± 0.10 (92.6%)
RIL127	19	3.16±0.68	0.17 ± 0.05	1191 ± 211	134 ± 31	1.05 ± 0.43	2.11 ± 0.55 (121.6%)
RIL149	20	5.23±0.95	0.24 ± 0.07	1271 ± 264	161 ± 30	1.63 ± 0.42	1.76 ± 0.11 (103.3%)
RIL200	23	7.21±1.86	0.27 ± 0.08	1247 ± 457	152 ± 40	2.01 ± 0.69	6.72 ± 1.23 (116.2%)

*N*: number of roots, RFW: root fresh weight, RDW: root dry weight, RL: root length, RS: root surface, RV: fine root volume.

The data represent all the roots of the non-inhibited plants (control) and inhibited plants (HgCl_2_). Values are means and standard deviations.

^a^Fine roots are the smallest diameter class (0 – 0.5 mm) determined by the WinRHIZO analysis.

^b^K^+^ fluxes are for HgCl_2_ treated plants but are also expressed in percentage of the controls (values in parenthesis).

### Variation of root hydraulic properties

**Figure 1:**
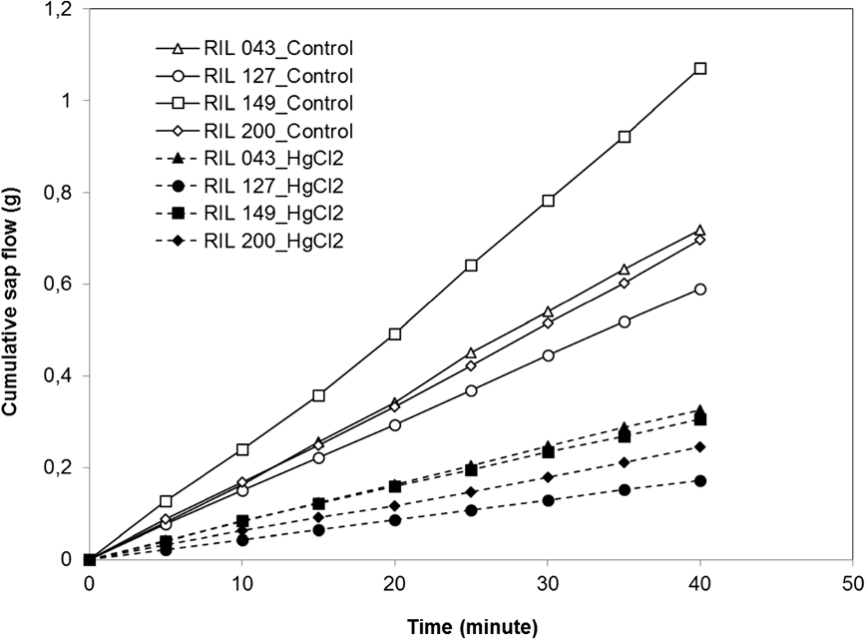
**The figure shows an example of cumulative sap flow for one root system per RIL and per treatment (open symbols for control and solid symbols for HgCl**_**2**_**) of RIL 043, RIL 127, RIL 149, RIL 200.** The cumulative data were obtained from eight successive, five minutes apart.

Figure 1 shows a representative time course of cumulative water movement of a root system before (control) and after treatment with HgCl_2_ for the four sunflower RILs. Sap flow (Jv) from untreated or treated root systems remained virtually constant throughout the 40 min measurement period. The ranking of RILs for Jv in untreated root systems was RIL149 > RIL43 = RIL200 > RIL127. Pressure-induced sap flow was almost twice as high in RIL 149 as in RIL 127. The sap flow was strongly inhibited by HgCl_2_ treatment in all genotypes (more than 50%).

Mean values of *L*_*0*_ and *Lp*_*r*_ showed significant variation between the four RILs. The values ranged from 0.7 to 1.2 μL s^-1^ MPa^-1^ and 5.10^-8^ to 8.10^>-8^ m s^>-1^ MPa^-1^, respectively. The ranking of RILs for *L*_*0*_ was RIL 149 > RIL 043 > =RIL 200 > RIL 127 but the ranking was changed for *Lp*_*r*_: RIL 149 > RIL 200 > RIL 127 > RIL 043. Differences between extreme values were above 60% in both cases.

### Contribution of AQPs to water uptake

**Figure 2:**
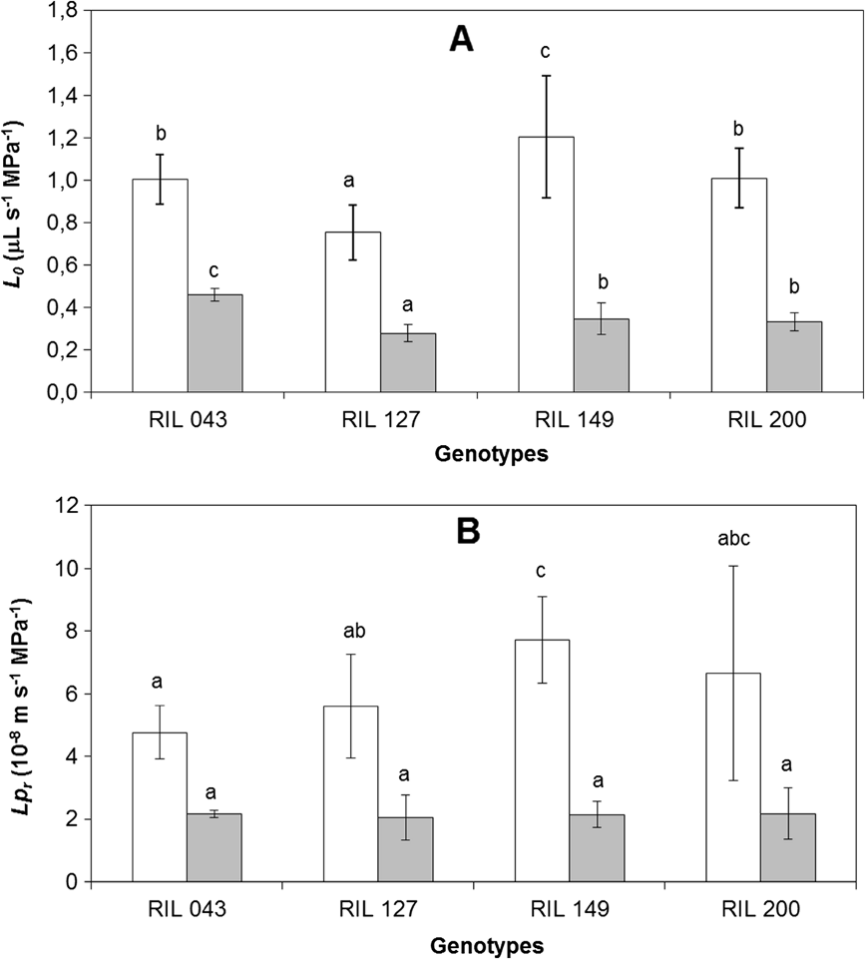
**Means of** ***L***_***0***_**(A) and** ***Lp***_***r***_**(B) of RIL 043, RIL 127, RIL 149, RIL 200.** The white bars are non-inhibited plants (control) and the black bars are inhibited plants (HgCl_2_). Each value is the mean of nine plants ± standard deviation. Means within a treatment without a common letter are significantly different by LSD_0.05_ test.

In our experiment, the contribution of AQPs to sap flow was explored using mercuric chloride. *L*_*0*_ and *Lp*_*r*_ fell to 30–40% of the control value and differences appeared between RILs (Figure 2A and B). HgCl_2_-treated root systems displayed markedly different *L*_*0*_ values between genotypes (Figure 2A) which ranked as follows: RIL 043 > RIL 149 > =RIL 200 > RIL 127 (*L*_*0*_ for RIL 043 was about 70% higher than for RIL 127). By contrast, *Lp*_*r*_ values were similar for all four RILs following HgCl_2_ treatment (Figure 2B).

**Figure 3:**
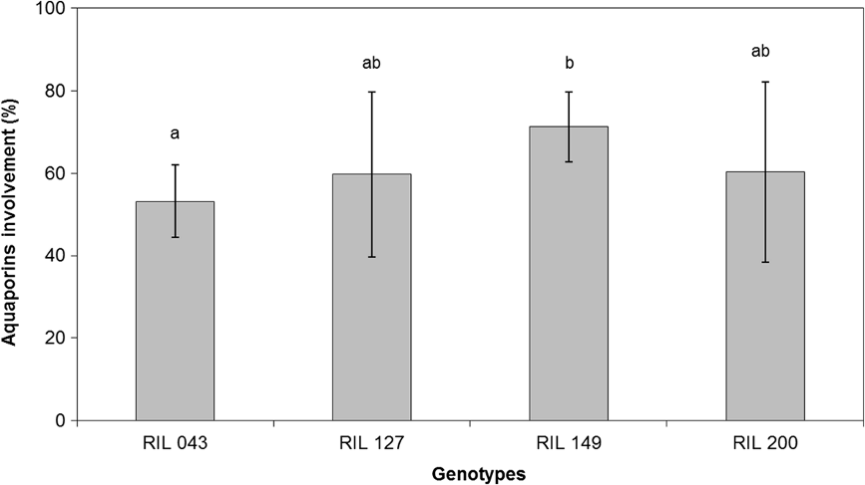
**Means of involvement of aquaporins of RIL 043, RIL 127, RIL 149, RIL 200.** Each value is the mean of nine plants ± standard deviation. Means without a common letter are significantly different by LSD_0.05_ test.

The contribution of AQPs to *Lp*_*r*_ (AQP involvement) expressed as the relative decrease in *Lp*_*r*_ induced by HgCl_2_ treatment was the highest in RIL 149 (73%) and the lowest in RIL 043 (55%) while other RILs displayed an intermediate contribution (Figure 3).

The flux of K^+^ into the xylem was not significantly affected by the presence of HgCl_2_ corresponding to 92.6%, 121.6%, 103.3% and 116.2% of the controls for RIL 043, RIL 127, RIL 149 and RIL 200, respectively (Table 1).

## Discussion

In this study, the values of *Lp*_*r*_ for sunflower root systems ranged from 5.10^-8^ to 8.10^-8^ m s^-1^ MPa^-1^ and were within the range of values reported for other species (Liu et al. [2009]; Sutka et al. [2011], Sakurai-Ishikawa et al. [2011]), although rather at the lower end of the *Lpr* scale reported for roots of annual (crop) plants. Our values for sunflower root hydraulic conductivity were very similar to those reported for roots of 20-d-old sunflower plants (*ca* 600 μL h^-1^ g^−1^ root fresh weight MPa^-1^, see Figure 1 and Table 1, Quintero et al. [1999]) but Alfalfa had *Lp*_*r*_ values 10 times higher than sunflower (Li et al. [2007]). The pressure that induces flow through root systems has been considered by some authors (Li et al. [2007]; Liu et al. [2009]) to be inappropriate to characterize “absolute” hydraulic values, because the externally applied pressure can induce flow through pathways external to the root system. Indeed, pressure chamber experiments give higher values of *Lp*_*r*_ than other methods using root pressure probes: resistance may vary according to the nature of the driving force for water movement (osmotic versus hydraulic) and the flow rate (Liu et al. [2009]). It has been shown by Vandeleur et al. ([2014]) that shoot manipulation affected root hydraulic characteristics in grapevine, soybean and maize. Here, we used detopped plants to measure root hydraulics, thus a note of caution is warranted regarding the absolute values reported. However, the purpose of the present work was to compare the hydraulics of several sunflower RILs differing in their whole-plant water relationships. Root *Lp* has been repeatedly shown to change with the volume flow rate through the root system (Sakurai-Ishikawa et al. [2011], Laur and Hacke [2013]). However, the flow rates induced here through pressure gradient across isolated root systems were similar to transpirational water loss rates of intact plants of the same age (Adiredjo et al. [2014]).

Mercuric chloride-induced reversible inhibition of root water flow is consistent with the presence of a protein-mediated path for trans-membrane sap flow in the sunflower root. To avoid non-specific effects, concentrations have to be as low as possible and exposure time as short as possible. Coskun et al. ([2012]) recommended caution when using aquaporin inhibitors including Hg^2+^. They showed membrane damage resulting from 500 μM Hg^2+^. Here we used the same relatively high HgCl_2_ concentration (500 μM). However, this value was similar to concentrations used in previous studies on whole root systems (Maggio and Joly [1995]; Peyrano et al. [1997]; Shimizu et al. [2005]; Ruggiero et al. [2007]). In addition, considering that we worked with sand (in which root excretion of organic compounds creates an organic matrix) and not in a hydroponics set-up, the effective concentration in the root zone was probably far below 500 μM due to immobilization of part of the Hg^2+^ by the system (Ruggiero et al. [2007]). The concentration used in this study was chosen from the preliminary dose response curves performed to identify a threshold concentration that had a marked effect on sap flow (for instance, 50 μM HgCl_2_ did not induce any depressive effect on sap flow) but that did not cause apparent irreversible toxicity effects. Indeed, the relationship between sap flow and applied pressure was highly linear suggesting that the Hg treatment did not cause broadly deleterious changes in root function during the time course of the pressure flow procedure (Maggio and Joly [1995]). In addition, there was no significant difference between control and HgCl_2_-treated roots in the amount of K^+^ recovered in the xylem exudates delivered through whole-root systems demonstrating that the Hg^2+^ concentration and exposure durations used here did not poison root cells in a way that might cause them to become leaky to ions (Maggio and Joly [1995]). Another convincing argument concerning the lack of general toxicity is the reversal of mercuric chloride inhibition by the scavenger 2-mercaptoethanol which is assumed to remove Hg from membranes of treated roots (Barrowclough et al. [2000]). Inhibition of sap flow by HgCl_2_ was reversed by *ca* 90% following rinsing of the root system in a mercaptoethanol solution (10 mM). In addition, sap continued to be spontaneously expressed from cut stems of excised roots several hours after mercury application demonstrating the generation of strong root pressure in the treated roots. Altogether these results indicate that HgCl_2_ did not reduce sap flow by a general inhibition of root metabolism, but rather by a direct effect on AQPs.

Leaves need to be continuously supplied with water and carbon dioxide to fulfill their photosynthetic function. The water transport capacity of the root (*L*_*0*_, root hydraulic conductance) is thus a key physiological parameter for whole-plant function since it determines the interplay between sap flow intensity and water potential gradients between soil and leaves. Differences in whole *L*_*0*_ reached a high value of 60% between sunflower genotypes. *L*_*0*_ was highest for RIL 149 and lowest for RIL 127 while RIL 043 and RIL 200 had similar intermediate values. *L*_*0*_ reflects the overall water uptake capacity of the root and results from both the root exchange surface area and its intrinsic water transport capacity (*Lp*_*r*_). *Lp*_*r*_ had a different ranking from *L*_*0*_, with the highest value for RIL 149 (as observed for *L*_*0*_) but the lowest for RIL 043 (whereas it was in second position for *L*_*0*_). *Lp*_*r*_ in RIL 049 was found to be 70% higher than in RIL 043. *Lp*_*r*_ was expressed in relation to the whole root area, assuming that outer cell layers were consistently representative of the hydraulic properties of all the segments of the root system (Sutka et al. [2011]). We also checked that when *Lp*_*r*_ was expressed in relation to root length or fine root volume, a similar difference between RIL 149 and RIL 043 was observed (see Table 1). Thus, genotype (RIL 043) can display both high whole root capacity (*L*_*0*_) and small intrinsic root capacity (*Lp*_*r*_). Sutka et al. ([2011]) described a substantial (2-fold) genetic variation in *Lpr*, establishing that *Arabidopsis* root hydraulic properties are far from uniform between natural accessions. We show here that large variations in root hydraulics also appear between sunflower genotypes.

*Lp*_*r*_ was sensitive to brief treatment with HgCl_2_. This allows *Lp*_*r*_ to be divided into two components: cell to cell and apoplastic pathways (ignoring the dilution-diffusion process across the double layer of membrane lipids which is not sensitive to mercury). The *Lp*_*r*_ of apoplasmic pathways, i.e. *Lp*_*r*_ measured in HgCl_2_, was identical for all genotypes. In other words, the conductance of the AQP-independent pathway (on an area basis) was similar for all RILs. Tissue mass, organization and/or cell wall structure (suberization of apoplastic barriers usually associated with root maturation which reduces water uptake capacity) may affect intrinsic root hydraulics. However, Sutka et al. ([2011]) reported that *Arabidopsis* accessions did not show any clear link between root suberization and the hydraulic conductivity of the AQP-independent path. In the present work, although sunflower genotypes displayed evident variations in root anatomy, no variations were observed in “intrinsic” apoplastic conductivity (*Lp*_*r*_ in HgCl_2_ treated roots). This suggests that the cell-to-cell pathway (aquaporin-dependent) was the major determinant of the “intrinsic” water transport properties of the organ (*Lp*_*r*_ in control plants),RIL 149 showing the highest involvement of AQPs (72%) and RIL 043 the lowest (55%). The relative contribution of AQPs to root conductivity (average 60% in the present experiment with sunflower) was similar to other estimates obtained in herbaceous species (Maggio and Joly [1995]; Tazawa et al. [1997]; Carvajal et al. [1999]; Barrowclough et al. [2000]; Shimizu et al. [2005]; Sutka et al. [2011]; Ruggiero et al. [2007]) confirming that pathways not involving AQPs can make a significant contribution to *Lp*_*r*_ (around 40%).

In the present study, sunflower genotypes were selected because of their contrasting behaviour with respect to water under well-watered conditions (Adiredjo et al. [2014]). A variety of hydraulic profiles can be observed between the four sunflower genotypes. RIL 149 and RIL 043 had the highest *L*_*0*_ but exhibited interesting and differing root properties. It appears that a “large” root anatomy (i.e. large root surface area, volume, and mass) allows RIL 043 to compensate for its lower contribution of AQPs to root hydraulics and therefore its lower *Lp*_*r*_. Whole *L*_*0*_ was only slightly lower in RIL 043 than in RIL 149, which had a greater *L*_*0*_ and the greatest *Lp*_*r*_ due to high AQP involvement. By contrast, RIL 127 had the lowest whole *L*_*0*_ due to small root development, despite higher intrinsic *Lp*_*r*_ and contribution of AQPs than RIL 043. RIL 200 exhibited intermediate values for all parameters. Interestingly, the ranking of the RILs for *L*_*0*_ was the same as the ranking of the RILs for WUE determined in our previous study: RIL 149 > RIL 043 > RIL 200 > RIL 127 (Adiredjo et al. [2014]). Therefore, *L*_*0*_ is suggested to play a key role in sunflower water balance and WUE (Maurel [2007]; Sade et al. [2010]). AQPs are reported to be regulated by several stresses, particularly drought, and shoots transpiration (Martre et al. [2001], [2002]; Clarkson et al. [2000]; Martínez-Ballesta et al. [2003]; Shimizu et al. [2005]; Sakurai-Ishikawa et al. [2011]; Laur and Hacke [2013]; Chaumont and Tyerman [2014]) often without any change in root anatomy or morphology. Under stress conditions, RIL 043, which displays the highest water transport capacity of the whole organ due to extensive root development, could be less affected than RIL 149 which presents high water transport capacities that depend on the contribution of AQPs.

## Conclusions

Three main conclusions emerge from our results: (i) a large variation occurs in morphological and hydraulic profiles in sunflower, (ii) there is a varying contribution of AQPs to hydraulic conductivity but a similar root intrinsic water permeability (*Lp*_*r*_, on an area basis) between genotypes and (iii) root anatomy, which appears to be a major determinant of the water transport properties of the whole organ, is able to compensate for a low AQP contribution.

## Authors’ original submitted files for images

Below are the links to the authors’ original submitted files for images. Authors’ original file for figure 1 Authors’ original file for figure 2 Authors’ original file for figure 3
