# Supplementary figures and images for: Hydraulic conductivity and contribution of aquaporins to water uptake in roots of four sunflower genotypes
Source: Bot Stud. 2014 Oct 30;55:75. doi: 10.1186/s40529-014-0075-1 (PMC5430332; doi:10.1186/s40529-014-0075-1)

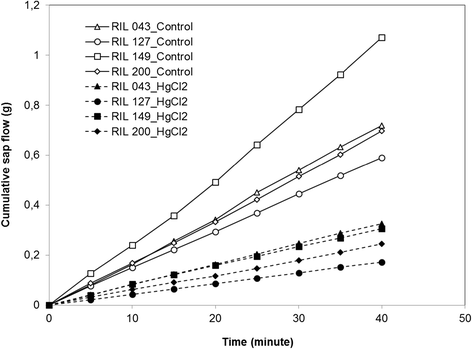

Supplement: Supplementary file 1 — Authors’ original file for figure 1 [file 40529_2014_9075_MOESM1_ESM.gif]

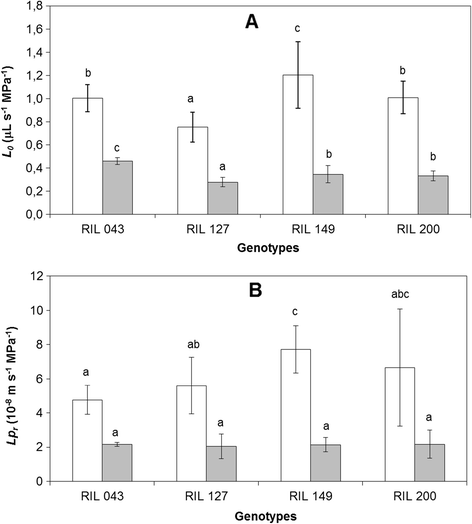

Supplement: Supplementary file 2 — Authors’ original file for figure 2 [file 40529_2014_9075_MOESM2_ESM.gif]

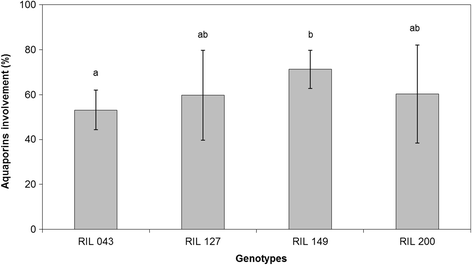

Supplement: Supplementary file 3 — Authors’ original file for figure 3 [file 40529_2014_9075_MOESM3_ESM.gif]
